# The Recovery After COVID-19 in Nursing Home Residents

**DOI:** 10.1177/23337214221094192

**Published:** 2022-04-11

**Authors:** Inge E. J. van der Krogt, Eefje M. Sizoo, Anouk M. van Loon, Simone A. Hendriks, Martin Smalbrugge

**Affiliations:** Department of Medicine for Older People, Amsterdam Public Health Research Institute, 1209 Amsterdam University Medical Centers Location VUmc, Amsterdam, The Netherlands

**Keywords:** COVID-19, nursing home residents, trajectory of disease, recovery, long-term care

## Abstract

**Introduction:**

Many nursing homes (NHs) are affected by COVID-19 and 30-day mortality is high. Knowledge on recovery of NH residents after COVID-19 is limited. Therefore, we investigated the trajectory in the first three months after a COVID-19 infection in NH residents.

**Methods:**

Retrospective observational cohort study of Dutch NH residents with COVID-19 between 1 September 2020 and 1 March 2021. Prevalence of COVID-19 symptoms and functioning was determined using interRAI (ADL-Hierarchy Scale (ADL-HS), Cognitive Performance Scale (CPS) and Revised Index of Social Engagement (RISE)) at four time points. Descriptive and pattern analyses were performed.

**Results:**

Eighty-six residents were included. Symptom prevalences after three months were higher than at baseline. At group level, functioning on all domains deteriorated and was followed by recovery towards baseline, except for ADL functioning. There were four trajectories; 9.3% had no deterioration. Total and partial recovery occurred in respectively 30.2% and 55.8% of the residents. In 4.7% there was no recovery.

**Conclusion:**

In 86% of NH residents surviving three months after COVID-19, occurrence of COVID-19 symptoms and deterioration in functioning was followed by recovery. COVID-19 symptoms fatigue and sleeping behaviour were significantly more prevalent, and ADL functioning was significantly lower, at three months compared to baseline.

## Introduction

On the 31st December 2019, the first worldwide case of COVID-19 was reported (World Health Organization, 2021). Since then, many nursing homes (NHs) have been affected by COVID-19 outbreaks. In the vulnerable NH population, the pandemic caused almost six times more deaths in the first month after the infection (Mas Romero et al., 2020). Rutten, van Loon, van Kooten, et al. (2020) described symptomatology and mortality of COVID-19 in NH residents in the first wave and showed that 63% experienced fever, 30% had dyspnoea, 63% developed a cough and 29% experienced atypical symptoms (delirium, confusion and sleepiness). The thirty-day mortality rate of symptomatic COVID-19 positive residents was 42%, with male gender, dementia, reduced kidney function and Parkinson’s Disease as risk factors. A Swedish study showed a comparable thirty-day mortality rate of 39.9% (Ballin et al., 2021). In the second wave, at the time of contact-tracing and active testing policies in NHs, thirty-day mortality rates were 22% (van Loon et al., 2021).

There is an increasing amount of data about the trajectory after COVID-19. Some patients develop long-lasting symptoms such as fatigue, dyspnoea, myalgia, headache, palpitations, amnesia and depression (Lopez-Leon et al., 2021). For NH residents, Rutten, van Loon, Joling, et al. (2020) showed that 46% experienced deteriorations before they improved and 54% recovered without fluctuations. However, the median follow-up time in this study was only ten days.

Yet, there is no data available on the long-term disease trajectory after COVID-19 for surviving NH residents. According to Lynn and Adamson’s trajectories of chronic illness, it is assumed that NH residents do not recover to pre-existing functioning after a COVID-19 infection (Lynn & Adamson, 2003). Their model of the trajectory of functioning in frailty shows that frail older adults are, in time, likely to slowly dwindle. Any intercurrent disease (e.g. COVID-19) may accelerate this decline in functioning. Whether, and to what extent COVID-19 indeed impacts functioning is important to know for proper prognostication.

The aim of this study is therefore to describe the trajectory of symptoms and functioning of NH residents in the first three months after a COVID-19 infection. We are particularly interested in cognition, ADL functioning (activities of daily living) and social functioning. Our hypothesis is that in general the NH residents will recover from the acute symptoms but will not completely recover to their pre-existing level of functioning.

## Methods

### Study and Patient Population

We conducted an observational retrospective cohort study. The study population contained Dutch NH residents with somatic and/or psychogeriatric conditions. Residents were eligible if they had: (a) an indication for long-term care, meaning they are residing in the NH; (b) a positive polymerase chain reaction (PCR) test for COVID-19 in the period between the 1^st^ of September 2020 and the 1^st^ of March 2021; and (c) were alive four weeks after the PCR-test. Exclusion criteria were residents in the dying phase, residents having an indication for palliative terminal care and admission to the NH less than one month before the positive PCR-test. In the above-mentioned period, residents were either tested with a PCR-test based on developing COVID-19 symptoms or based on contact-tracing.

Eligible residents (or their legal representative in case the NH resident was incompetent to make the decision) were approached by elderly care physicians (ECPs) in training. ECPs are medical specialists responsible for the medical care of NH residents in the Netherlands (Koopmans et al., 2017). ECPs in training approached the positive tested NH residents, starting with the most recent, until they reached a maximum of ten residents per ECP in training.

### Ethics

Written informed consent was obtained from the resident or legal representative. Approval by The Medical Ethics Review Committee of Amsterdam UMC was granted (2020.0694). The Medical Research Involving Human Subjects Act does not apply to this study protocol.

### Data Collection

Two consecutive groups of ECPs in training, working approximately 8 months in the facility, gathered the data as part of their education programme. Together with nursing staff involved in daily care of the NH residents, they filled in a case report form (CRF) that consisted of questions about age, gender, estimated weight, vaccination status, co-morbidities, COVID-19 symptoms and cognition, ADL functioning and social functioning. Patient files were checked for medical history and for additional information on functioning and symptoms.

The CRF was filled in for four different time points, all retrospectively. These four time points were defined as: the month prior to the positive PCR-test (T=0), the worst moment of the infection in the first month (T=1), one month after the PCR-test (T=2) and three months after the PCR-test (T=3).

### Measurements

#### Questionnaire

The used questionnaire was composed about main COVID-19 symptoms and for domains of functioning of questions derived from the validated interRAI-questionnaire (http://www.interrai.org), see below. InterRAI includes questionnaires and algorithms, which provide reliable and validated signals and indications for, among others, medicine for older adults. The InterRAI has a good reliability (Cronbach’s alpha of >0.75) (Kim et al., 2015). This questionnaire is already being used in some NHs to explore the problems and needs of the older adults in a structured manner so that all care providers have a clear view of the resident (InterRAI, 2021).

#### COVID-19 symptoms

We evaluated the presence of the COVID-19 symptoms of fatigue, sleeping behaviour, cough, dyspnoea and loss of smell and/or taste. For evaluation of fatigue (ordinal scale 0–4), sleeping behaviour (ordinal scale 0–3) and dyspnoea (ordinal scale 0–3), we used the existing InterRAI items. We added cough on an ordinal scale (0 absent, 1 mild, 2 moderate and 3 severe) and loss of smell and/or taste on a dichotomous scale (0 absent, 1 present). The ordinal scales (fatigue, sleeping behaviour, cough and dyspnoea) were dichotomised into present (≥1) or absent (0) in order to have a clearer overview of the existing symptoms at the different time points.

#### Functioning domains

ADL functioning was determined using the ADL Hierarchy Scale (ADL-HS) from InterRAI (InterRAI, 2021). This measurement is ranked on a seven-point-scale (0–6)*: ‘Independent’, ‘Supervision’, ‘Limited assistance’, ‘Extensive assistance’, ‘Maximal assistance’, ‘Dependent’ and ‘Total dependent’.* To determine the score of each resident, a decision tree with questions about toilet use, personal hygiene, locomotion and eating was used. Loss of ADL functions with severe impairments, such as eating, causes a higher score of dependence compared to functions associated with less severe impairments (Morris et al., 1999).

In order to assign a score to the cognitive functioning of each resident, we used the Cognitive Performance Scale (CPS) from InterRAI (InterRAI, 2021). The CPS is based upon the answers on the questions about decision making, eating, short-term memory and making themselves understood. The CPS is ranked on a seven-point-scale (0–6): *‘Intact’, ‘Borderline intact’, ‘Mild impairment’, ‘Moderate impairment’, ‘Moderately severe impairment’* and *‘Very severe impairment’* (Morris et al., 1994).

To score social engagement we used the Revised Index of Social Engagement (RISE) from InterRAI (InterRAI, 2021). The RISE was calculated with six items of the CRF that were related with social engagement: - interacting with others, - doing planned or structured activities, - accepting invitations to most group activities, - pursuing involvement in life of facility, - initiating interactions with others and - reacting positively to interactions initiated by others. The RISE is a 0–6 point scale, indicating the level of social engagement, where higher scores mean more social engagement in the NH (Yoon & Kim, 2017).

### Data Analysis

#### Descriptive analyses

Patient characteristics at baseline were described with descriptive statistics. For symptom prevalence, we calculated frequencies and percentages. Means and standard deviations of the ADL-HS, RISE and CPS scores over the different time points were calculated. The data was analysed in Statistical Package for the Social Sciences (SPSS) version 26.

#### Pattern analyses

**Figure 1.**
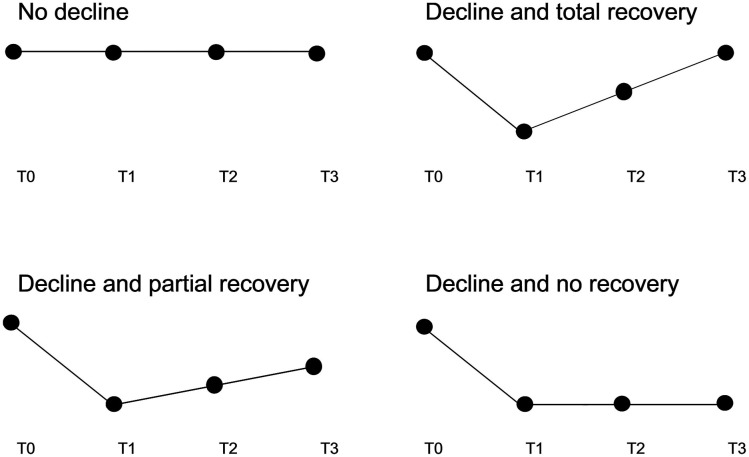
Different trajectories of COVID-19 symptoms and functioning domains

Since we measured at four time points, we had four values (corresponding with the answer possibilities of the questionnaires) for each variable (symptoms and domains of functioning). Next, we described the observed patterns of these four values as trajectories for each symptom and domain of functioning for each individual resident separately. These different trajectories were categorized as: ‘no decline’, ‘decline and total recovery’, ‘decline and partial recovery’ and ‘decline and no recovery’ (see Figure 1). A decline for a resident was defined as a deterioration of at least 1 point on the associated questionnaire at time point T1 (the second time point in the pattern) compared to T0 (the first time point in the pattern). ‘No decline’ consisted of patterns that either show no deterioration over the study period, show an improvement over T1 or show a deterioration that started at T2 or T3 (for example respectively; 2-2-2–2, 2-1-2–2, 1-1-2–2). The latter to relate the deterioration to the COVID-19 infection. An exception was made for fatigue and sleeping behaviour because we argued that these complaints often emerge later in time after an COVID-19 infection. Thus, sleeping behaviour and fatigue patterns that show an increase at T2 or T3 were included in the other trajectories categories. ‘Decline and total recovery’ contained all the patterns that returned to baseline level after a deterioration at T1 (e.g. 1-3-2–1). ‘Decline and partial recovery’ consisted of the patterns were some recovery had taken place but where it did not return to baseline level after three months (e.g. 1-3-2–2). The last category, ‘Decline and no recovery’, meant that the increase in symptom burden or deterioration in functioning at T1 remained present at the same level after three months (e.g. 1-3-3–3). Next, the prevalence of the four different trajectories was calculated per variable (fatigue, sleeping behaviour, cough, dyspnoea, loss of smell and/or taste, ADL-HS, CPS and RISE).

To show how many residents are ‘back to their old self’ after three months, we also described the trajectories for all COVID-19 symptoms combined, for the three functioning domains combined and for all COVID-19 symptoms and three functioning domains combined.

### Statistical Analyses

For differences in symptomatology between T0 (before infection) and T3 (three months after infection) we used the McNemar test to evaluate changes over time. For the difference in the function domains before and three months after the COVID-19 infection (T0 vs. T3) we used the Wilcoxon signed rank test. We applied Bonferroni correction because of multiple comparisons.

## Results

### Resident Characteristics

In total, 86 residents participated. All residents were included three months after the infection. Baseline characteristics are described in Table 1. The mean age of the NH residents was 84 years (SD 8) and they were mostly women (76.7%). Dementia was diagnosed in 72.1% of the residents, and Alzheimer’s dementia was most common (46.8%). Eight residents included by the second group of ECPs received their first vaccination before they got infected (median of 5 days after the first vaccination), no one was fully vaccinated before the infection. Apart from this, we found no statistically significant differences in between the residents included by the two consecutive groups of ECPs (data not shown).

**Table 1.** Baseline characteristics.

Nursing Home Residents (*N*=86)		(*N*=86)
Age		Mean 84 (SD 8)
Gender	Female	66 (76.7%)
Estimated weight	Underweight	7 (8.1%)
	Normal weight	56 (65.1%)
	Overweight	23 (26.7%)
Dementia		62 (72.1%)
*Type of dementia*	*Alzheimer’s*	29 (46.8%)
	*Vascular*	8 (12.9%)
	*Mixed (Alzheimer’s and vascular)*	10 (16.1%)
	*Lewy Body Dementia*	0 (0.0%)
	*Parkinson’s Dementia*	1 (1.6%)
	*Unspecified*	14 (22.6%)
*Severity of dementia*	Mild (GDS* 1 t/m 4)	1 (1.6%)
	Moderate (GDS* 5 of 6)	41 (66.1%)
	Severe (GDS* 7)	20 (32.3%)
Psychiatric disease		27 (31.4%)
Cardiovascular disease (incl. CVA)		60 (69.8%)
Diabetes Mellitus		10 (11.6%)
Renal insufficiency (eGFR <60)		19 (22.1%)
Pulmonary disease		17 (19.8%)
Neurological disease (excl. CVA and dementia)		18 (20.9%)

(*) GDS: Global Deterioration Scale

### Time Points

For half of the study population (*N*=45), patients included by the second group of ECPs in training, data was available about exact dates related to the different time points. T0 was on average 19 days before the PCR-test, T1 was 3 days after the PCR test, between T1 and T2 were on average 29 days and between T2 and T3 were on average 60 days.

### COVID-19 Symptoms

**Figure 2.**
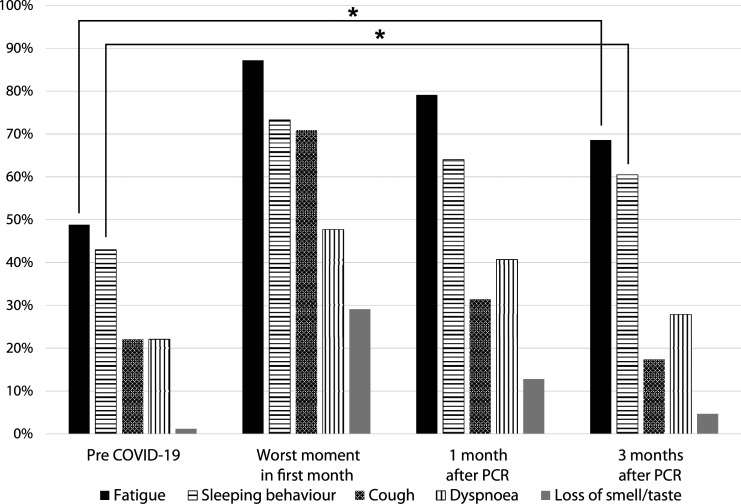
Prevalence (%) of COVID-19 symptoms. (*) Statistically significant difference.

For the total group, prevalences of the different symptoms on the four time points are shown in Figure 2 (see supplemental A for the corresponding table with exact percentages). For ‘loss of smell and/or taste’, there were 15–23 cases of missing data depending on the time point, probably because dementia was common in this population. If we excluded the missing data, there was a chance of overestimation. Therefore we chose to interpret these as ‘not present’, with the chance of little underestimation. Many residents already suffered from various symptoms before they got infected with COVID-19, such as fatigue (48.8%), the need of one or multiple naps during the day (‘sleeping behaviour’) (43.0%) and coughing (22.1%). At the worst moment of the infection (T1), most residents (87.2%) suffered from fatigue, whereas the prevalence of coughing and the loss of smell and/or taste increased the most compared to baseline (respectively, from 22.1% to 70.9% and from 1.2% to 29.1%). Experienced fatigue and sleeping behaviour were still significantly increased after three months compared to baseline (respectively *p<0,001* and *p=0.001*), while dyspnoea, coughing and loss of smell and/or taste did not change significantly compared to baseline (respectively *p=0.180, p=0.125 and p=0.125*).

### ADL-functioning, cognition and social functioning

**Figure 3.**
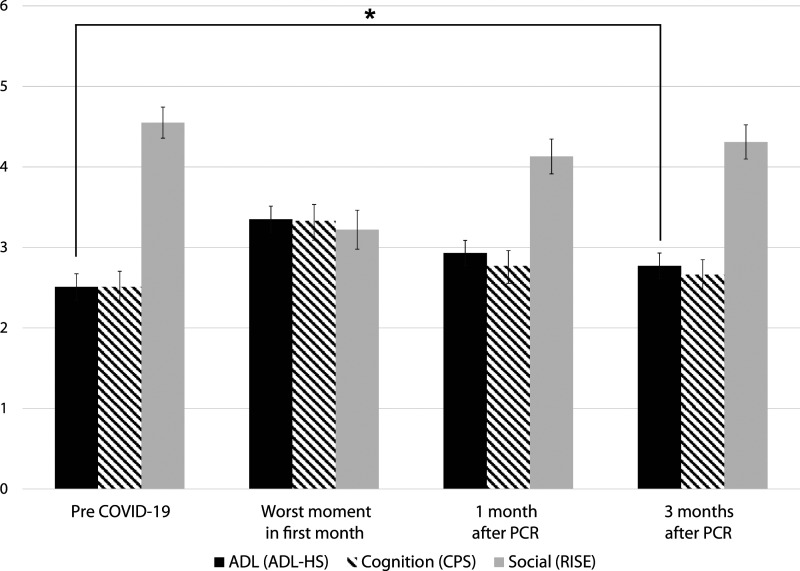
Mean (SE) of ADL-, cognitive- and social functioning. ADL-HS and CPS: higher mean means worse functioning. RISE: higher mean means better functioning. (*) Statistically significant difference

In Figure 3 the means of the ADL-HS, CPS and RISE are presented among the different time points (see also supplemental B). In general, the same pattern is observed in all three scores. A decline in functioning on T1 occurred (ADL-HS from 2.51 to 3.35, CPS from 2.51 to 3.33 and RISE from 4.55 to 3.22), after which the residents slowly returned towards baseline score after three months, with only a significant decline in ADL functioning compared to baseline (T0 vs. T3: ADL-HS 2.55 vs. 2.77 (*p=0.007*), CPS 2.51 versus 2.66 (*p=0.144*) and RISE 4.55 versus 4.31 (*p=0.054*)). We evaluated exploratively if dementia would influence this pattern. The overall pattern for the functional domains appeared to be similar (see supplemental C).

### Trajectories of Symptoms and Functioning

Frequencies of the different trajectories of symptoms and functions are presented in Tables 2 and 3. For different individual areas, many residents did not experience worsening of symptoms or a development of new symptoms (‘No decline’). This was especially the case for dyspnoea and loss of smell and/or taste (respectively 62.8% and 70.9%). Two-third (62.8%) of the residents developed new coughing complaints or an increase of pre-existing coughing complaints. All these residents fully recovered or recovered to baseline level after three months. Residents were less likely to recover from new fatigue complaints and changes in sleeping behaviour (19.8% and 17.4%) compared to the other symptoms (ranging from 0.0% to 4.7%). More than half of the residents did not develop a deterioration in ADL, cognitive or social functioning (respectively, 54.7%, 65.1% and 54.7%). Of the other half who did decline, most residents completely recovered and 14.0%–17.5% maintained a degree of deterioration in ADL, cognitive or social functioning after three months.

**Table 2.** Different trajectories of individual COVID-19 symptoms and function domains.

		COVID-19 symptoms					Function domains		
	**Trajectory % (n)**	Fatigue	Sleeping behaviour	Cough	Dyspnoea	Loss of smell and/or taste	ADL (ADL-HS)	Cognition (CPS)	Social (RISE)
	No decline 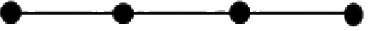	20.9% (18)	36.0% (31)	37.2% (32)	62.8% (54)	70.9% (61)	54.7% (47)	65.1% (56)	54.7% (47)
Decline	Total recovery 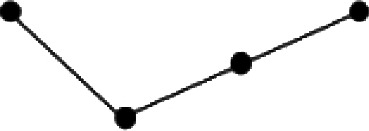	36.0% (31)	37.2% (32)	62.8% (54)	29.1% (25)	24.4% (21)	27.9% (24)	17.4% (15)	31.4% (27)
	Partial recovery 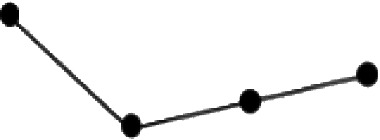	23.3% (20)	9.3% (8)	0	7.0% (6)	0	7.0% (6)	8.1% (7)	10.5% (9)
	No recovery 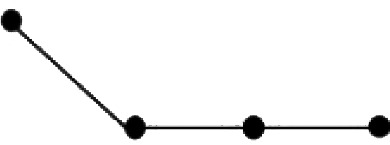	19.8% (17)	17.4% (15)	0	1.2% (1)	4.7% (4)	10.5% (9)	9.3% (8)	3.5% (3)

**Table 3.** Different trajectories of combinations of all COVID-19 symptoms and function domains.

	Trajectory % (n)	COVID-19 symptoms combined	Function domains combined	All five COVID-19 symptoms and all three function domains
	No decline 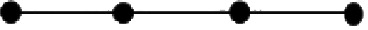	10.5% (9)	30.2% (26)	9.3% (8)
Decline	Total recovery 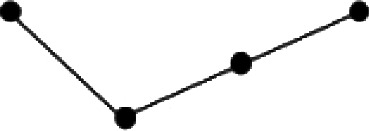	36.0% (31)	36% (31)	30.2% (26)
	Partial recovery 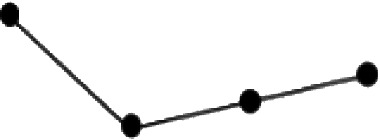	46.5% (40)	29.1% (25)	55.8% (48)
	No recovery 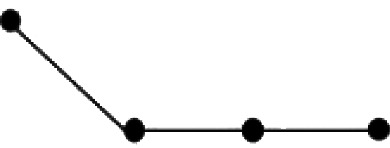	7% (6)	4.7% (4)	4.7% (4)

For the trajectory of all symptoms combined (see Table 3*)*, we observed that 10.5% of the residents did not experience worsening or new symptoms and 36.0% completely recovered from the developed or worsened symptom(s). Only 7% did not experience any recovery from all the developed or worsened symptoms at three months. Decline followed by total or partial recovery of the three function domains combined occurred in about one-third of the residents (36% and 29.1%, respectively). There was no deterioration in any function domain in 30.2% of the residents. When combining all eight areas (five symptoms and three function domains), eight residents (9.3%) experienced no deterioration at all. A degree of recovery after deterioration was found in 86% of the residents. In only 4.7% the total deterioration remained present after three months. There were no statistically significant differences in baseline characteristics between the residents who recovered and those who did not or only partially recovered (see supplemental D).

## Discussion

We investigated the trajectory of COVID-19 symptoms and of functioning domains in the first three months after a COVID-19 infection in NH residents who already survived the first three months. We observed that most residents who developed new or increased symptoms or deterioration in functioning (ADL, cognition, social) as a result of an infection with COVID-19, showed recovery after three months. After three months, the COVID-19 symptoms fatigue and sleeping behaviour were significantly more prevalent, and ADL functioning was significantly lower, compared to baseline. Yet, most of these residents did not recover to baseline functioning. With these findings, residents and relatives can be informed about the prognosis of COVID-19 infections and it can be used for advance care planning.

Regarding symptomatology of COVID-19 infections, another recent retrospective cohort study in adults (mean age of 46) with COVID-19 showed that 36.5% of them had at least one COVID-19 related feature reported in the 90–180 days after the diagnosis (Taquet et al., 2021). We found that more than half of the residents (53.5%) experienced at least one of the symptoms three months after the diagnosis. That we found a higher percentage in our study is probably because our population is older and more vulnerable.

As mentioned previously, according to the disease trajectories model of Lynn and Adamson (2003), natural deterioration over time is to be expected in frail elderly. Since this study has no control group, it is hard to say if the observed decline in functioning is related to COVID-19 or to natural course. Therefore we evaluated the different domains of functioning and compared this with other literature. Regarding cognitive functioning of the NH residents, we found that 17.4% had a decline in cognitive performance three months after the infection. This decline is in line with a study that investigated influencing factors of cognitive decline in newly placed NH residents with mixed comorbidities where cognitive decline was present in 16% of the newly placed NH residents after three months (Freeman et al., 2017). This was also measured with the CPS and shows the natural course of cognitive functioning in NH residents after admission. The comparability of these results implies that the impact of a COVID-19 infection is comparable to the impact of admittance to a nursing home. When looking at ADL functioning, we found that 17.5% experienced decline in functioning after three months. This is in line with Binder et al. (2003), who showed that approximately 19% of the NH residents experienced ADL decline or died in the 90 days after a lower respiratory tract infection (LRI). Next, for social engagement, we found that after three months 14% of the residents still had decline in social engagement. An older study observed that 24% of the NH residents experienced a decline in social engagement over six months (Phillips et al., 1997). This is a higher proportion than our study (14%), but this is also measured over a longer period in time and thus representing natural course. In summary, the deterioration in the three domains that we see between the measurement just before the COVID-19 infection and three months thereafter appears to be partly caused by expected ‘natural’ deterioration and presumably partly caused by the infection given the similar patterns that occur in other intercurrent infections.

To our best knowledge, this is the first study investigating the trajectory of functioning and symptoms in the first three months after a COVID-19 infection in NH residents. A strong aspect is that we used observational data by nursing staff as a source of information, which allowed participation of the whole spectrum of NH residents, as residents in a poor condition were not bothered by questionnaires and interviews. This increased the likelihood of representative sampling. A limitation is that the data is obtained retrospectively by nursing staff. This might have caused recall bias. Worse functioning and severe symptoms stand out more and lead to a higher chance of overestimation. Yet, more subtle complaints and deterioration in function may be less well remembered and therefore are possibly underestimated. For the calculation of the mean days between the different time points, data for only half of the population was available because this question was added for the second group. We do not expect that the first group included differently since instructions on inclusion did not change. Indeed when tested the baseline characteristic did not differ between the two groups. For the prevalence of loss of smell and/or taste there were between 15 and 23 cases of missing data depending on the time point. We interpreted the missing data as ‘not present’. The actual prevalence of loss of smell and/or taste may be higher. When interpreting the results, we must be aware that we can only say something about the three months survivors of COVID-19, as we did not have residents that died between 1 month and 3 months after a positive PCR-test in our sample. Also, the reason of testing (based on developing symptoms or based on contact-tracing) might have an influence on the recovery. The study of Paap et al. (2021) showed that NH residents who were tested based on symptoms had a decreased chance of survival in the first 30 days compared to the NH residents who were tested based on contact-tracing.

For future research, it would be interesting to study the effect of vaccination status in NH residents. People who are infected after they are vaccinated develop less severe symptoms (Centers for Disease Control and Prevention, 2021). Perhaps, the vaccine will also accelerate or improve recovery in NH residents. Next, the reason of testing in relation to the recovery should be investigated. Lastly, it would be interesting to look at long-term outcomes to observe if partially recovered residents will continue recovering to baseline or that this is their ‘endpoint’.

## Conclusion

We have shown that the majority (86%) of the Dutch NH residents who have survived three months after the COVID-19 infection, had a trajectory in which a degree of recovery of occurred. Three months after the COVID-19 infection, 30.2% of the NH residents fully functioned again as they did before the infection. After three months, the COVID-19 symptoms fatigue and sleeping behaviour were significantly more prevalent. Mean ADL functioning was significantly deteriorated after three months compared to baseline. Mean cognitive functioning was unchanged, while a declining trend was seen in mean social functioning. These findings are important and can be used to inform residents and proxies about prognosis of COVID-19 infections and for advance care planning.

## Supplemental Material

sj-pdf-1-ggm-10.1177_23337214221094192 – Supplemental Material for The Recovery After COVID-19 in Nursing Home Residents Click here for additional data file. Supplemental Material, sj-pdf-1-ggm-10.1177_23337214221094192 for The Recovery After COVID-19 in Nursing Home Residents by Inge E. J. van der Krogt, Eefje M. Sizoo, Anouk M. van Loon, Simone A. Hendriks and Martin Smalbrugge in Gerontology and Geriatric Medicine

## References

[bibr1-23337214221094192] Ballin M. Bergman J. Kivipelto M. Nordström A. Nordström P. (2021). Excess Mortality After COVID-19 in Swedish Long-Term Care Facilities. J Am Med Dir Assoc, 22(8), 1574–1580.e1578. 10.1016/j.jamda.2021.06.010 34174196 PMC8223135

[bibr2-23337214221094192] Binder E. F. Kruse R. L. Sherman A. K. Madsen R. Zweig S. C. D'Agostino R. Mehr D. R. (2003). Predictors of short-term functional decline in survivors of nursing home-acquired lower respiratory tract infection. J Gerontol A Biol Sci Med Sci, 58(1), 60–67. 10.1093/gerona/58.1.m60 12560413

[bibr3-23337214221094192] Centers for Disease Control and Prevention. (2021). The possibility of COVID-19 after vaccination: Breakthrough infections*.* https://www.cdc.gov/coronavirus/2019-ncov/vaccines/effectiveness/why-measure-effectiveness/breakthrough-cases.html

[bibr4-23337214221094192] Freeman S. Spirgiene L. Martin-Khan M. Hirdes J. P. (2017). Relationship between restraint use, engagement in social activity, and decline in cognitive status among residents newly admitted to long-term care facilities. Geriatr Gerontol Int, 17(2), 246–255. 10.1111/ggi.12707 26822624

[bibr5-23337214221094192] InterRAI. (2021). Retrieved July 14, 2021 from https://interrai.org

[bibr6-23337214221094192] Kim H. Jung Y. I. Sung M. Lee J. Y. Yoon J. Y. Yoon J. L. (2015). Reliability of the interRAI long term care facilities (LTCF) and interRAI Home Care (HC). Geriatr Gerontol Int, 15(2), 220–228. 10.1111/ggi.12330 25163513

[bibr7-23337214221094192] Koopmans R. Pellegrom M. van der Geer E. R. (2017). The dutch move beyond the concept of nursing home physician specialists. J Am Med Dir Assoc, 18(9), 746–749. 10.1016/j.jamda.2017.05.013 28668662

[bibr8-23337214221094192] Lopez-Leon S. Wegman-Ostrosky T. Perelman C. Sepulveda R. Rebolledo P. A. Cuapio A. Villapol S. (2021). More than 50 long-term effects of COVID-19: a systematic review and meta-analysis. Sci Rep, 11(1), 16144. 10.1038/s41598-021-95565-8 34373540 PMC8352980

[bibr9-23337214221094192] Lynn J. Adamson D. (2003). Living well at the end of life: Adapting health care to serious chronic illness in old age. Rand Health.

[bibr10-23337214221094192] Mas Romero M. Avendaño Céspedes A. Tabernero Sahuquillo M. T. Cortés Zamora E. B. Gómez Ballesteros C. Sánchez-Flor Alfaro V. López Bru R. López Utiel M. Celaya Cifuentes S. Peña Longobardo L. M. Murillo Romero A. Plaza Carmona L. Gil García B. Pérez Fernández-Rius A. Alcantud Córcoles R. Roldán García B. Romero Rizos L. Sánchez Jurado P. M. León Ortiz M. Abizanda P. (2020). COVID-19 outbreak in long-term care facilities from Spain. Many lessons to learn. PLoS One, 15(10), Article e0241030. 10.1371/journal.pone.0241030 33108381 PMC7591018

[bibr11-23337214221094192] Morris J. N. Fries B. E. Mehr D. R. Hawes C. Phillips C. Mor V. Lipsitz L. A. (1994). MDS cognitive performance scale. J Gerontol, 49(4), M174–M182. 10.1093/geronj/49.4.m174 8014392

[bibr12-23337214221094192] Morris J. N. Fries B. E. Morris S. A. (1999). Scaling ADLs within the MDS. J Gerontol A Biol Sci Med Sci, 54(11), M546–M553. 10.1093/gerona/54.11.m546 10619316

[bibr13-23337214221094192] Paap K. C. van Loon A. M. van Rijs S. M. Helmich E. Buurman B. M. Smalbrugge M. Hertogh C. (2021). Symptom- and prevention-based testing of COVID-19 in nursing home residents: A retrospective cohort study. Gerontol Geriatr Med, 7(1), 23337214211055338. 10.1177/23337214211055338 34790840 PMC8591646

[bibr14-23337214221094192] Phillips C. D. Morris J. N. Hawes C. Fries B. E. Mor V. Nennstiel M. Iannacchione V. (1997). Association of the resident assessment instrument (RAI) with changes in function, cognition, and psychosocial status. J Am Geriatr Soc, 45(8), 986–993. 10.1111/j.1532-5415.1997.tb02971.x 9256853

[bibr15-23337214221094192] Rutten J. J. S. van Loon A. M. Joling K. J. Smalbrugge M. van Buul L. W. Hertogh C. (2020). [COVID-19 in nursing homes A study of diagnosis, symptomatology and disease course]. Ned Tijdschr Geneeskd, 164(1), D5173. (Covid-19 in verpleeghuizen.) 32779925

[bibr16-23337214221094192] Rutten J. J. S. van Loon A. M. van Kooten J. van Buul L. W. Joling K. J. Smalbrugge M. Hertogh C. (2020). Clinical Suspicion of COVID-19 in nursing home residents: symptoms and mortality risk factors. J Am Med Dir Assoc, 21(12), 1791–1797. 10.1016/j.jamda.2020.10.034 33256958 PMC7598901

[bibr17-23337214221094192] Taquet M. Dercon Q. Luciano S. Geddes J. R. Husain M. Harrison P. J. (2021). Incidence, co-occurrence, and evolution of long-COVID features: A 6-month retrospective cohort study of 273,618 survivors of COVID-19. PLoS Med, 18(9), Article e1003773. 10.1371/journal.pmed.1003773 34582441 PMC8478214

[bibr18-23337214221094192] van Loon A. M. Rutten J. J. S. van Buul L. W. van Kooten J. Joling K. J. Smalbrugge M. Hertogh C. (2021). Factsheet 4: Morbiditeit en mortaliteit 1e vs. 2e golf*.* https://unoamsterdam.nl/wp-content/uploads/2021/01/20210219Factsheet-4-1e-vs-2e-golf.pdf

[bibr19-23337214221094192] World Health Organization. (2021). Coronavirus disease (COVID-19) pandemic*.* https://www.euro.who.int/en/health-topics/health-emergencies/coronavirus-covid-19/novel-coronavirus-2019-ncov 37184163

[bibr20-23337214221094192] Yoon J. Y. Kim H. (2017). The revised index for social engagement in long-term care facilities: A psychometric study. Journal of Nursing Research, 25(3), 216–223. 10.1097/jnr.0000000000000156 28481817

