# Supplementary material for: The Recovery After COVID-19 in Nursing Home Residents
Source: Gerontol Geriatr Med. 2022 Apr 11;8:23337214221094192. doi: 10.1177/23337214221094192 (PMC9005824; doi:10.1177/23337214221094192)
Supplement: sj-pdf-1-ggm-10.1177_23337214221094192 – Supplemental Material for The Recovery After COVID-19 in Nursing Home Residents [file sj-pdf-1-ggm-10.1177_23337214221094192.pdf]

Supplementary

Table of contents:

A. Prevalence of COVID-19 symptoms
B. Mean functioning of different domains (ADL, cognition, social)
C. Mean functioning of different domains (ADL, cognition, social) stratified for dementia
D. Statistical analysis of the differences in baseline characteristics between the different trajectories

Supplement A – Prevalence of COVID-19 symptoms

**Symptomatology % (n)**	**T0**	**T1**	**T2**	**T3**
Fatigue	48.8% (42)	87.2% (75)	79.1% (68)	68.6% (59)
Sleeping behaviour	43.0% (37)	73.3% (63)	64% (55)	60.5% (52)
Cough	22.1% (19)	70.9% (61)	31.4% (27)	17.4% (15)
Dyspnoea	22.1% (19)	47.7% (41)	40.7% (35)	27.9% (24)
Loss of smell/taste	1.2% (1)	29.1% (25)	12.8% (11)	4.7% (4)

Supplement B – Mean functioning of different domains (ADL, cognition, social)

**Functioning mean (SE)**	**T0**	**T1**	**T2**	**T3**
ADL (ADL-HS)	2.51 (0.16)	3.35 (0.16)	2.93 (0.16)	2.77 (0.16)
Cognition (CPS)	2.51 (0.19)	3.33 (0.20)	2.77 (0.19)	2.66 (0.19)
Social (RISE)	4.55 (0.19)	3.22 (0.24)	4.13 (0.22)	4.31 (0.21)

***Supplement C* – Mean functioning of different domains (ADL, cognition, social) stratified for dementia**

Supplement D - Statistical analysis of the differences in baseline characteristics between the different trajectories

To assess whether there were differences in baseline characteristics between the residents who were not or only partially recovered compared to residents who did recover, we ran some additional analyses. Given the relatively small sample size, we ran the analyses on the 'overall recovery' variable (all function domains and all symptoms combined). Since, there were only 4 residents who did not recover, we decided to combine this group with the partial recovery group (N=48) and compared this combined group (N=56) ('no recovery' & 'partial recovery') to the completely recovered group (N=26). We performed a chi-squared statistical test to evaluate significant baseline differences between the residents who recovered and those who did not or partially recovered. There were no significant differences between the residents. See the table below for the results.

		**No/Partial recovery**	**Recovery**	P-value (chi-squared)
		N = 52	N = 26	
Age		84 (SD 9)	82 (9)	0.383
Gender	Female	38 (73.1%)	20 (76.9%)	0.714
Estimated weight	Underweight	5 (9.6%)	1 (3.8%)	0.653
	Normal weight	33 (63.5%)	17 (65.4%)	
	Overweight	14 (26.9%)	8 (30.8%)	
Dementia		38 (73.1%)	18 (69.2%)	0.722
Type of dementia	Alzheimer's	20 (52.6%)	6 (33.3%)	0.210
	Vascular	3 (7.9%)	5 (27.8%)	

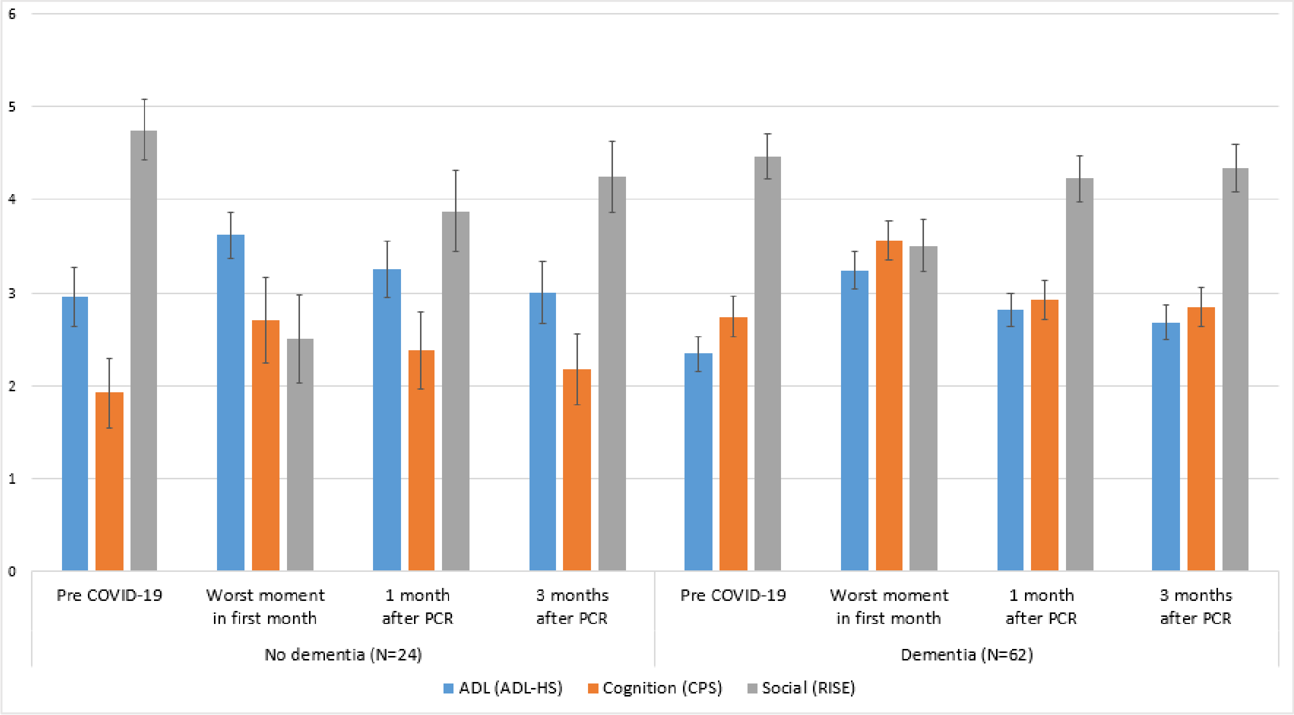

	Mixed	6 (15.8%)	2 (11.1%)	
	Lewy Body	0 (0%)	0 (0%)	
	Parkinson's	1 (2.6%)	0 (0%)	
	Unspecified	8 (21.1%)	5 (27.8%)	
Severity of dementia	Mild	1 (2.6%)	0 (0%)	0.161
	Moderate	22 (57.9%)	15 (83.3%)	
	Severe	15 (39.5%)	3 (16.7%)	
Psychiatric disease		16 (30.8%)	9 (34.6%)	0.732
Cardiovascular disease (incl. CVA)		35 (67.3%)	22 (84.6%)	0.104
Diabetes Mellitus		7 (13.5%)	3 (11.5%)	0.811
Renal insufficiency (eGFR <60)		9 (17.3%)	8 (30.8%)	0.175
Pulmonary disease		12 (23.1%)	5 (19.2%)	0.698
Neurological disease (excl. CVA and dementia)		11 (21.2%)	6 (23.1%)	0.864
